# A randomised, double-masked phase III/IV study of the efficacy and safety of Avastin^® ^(Bevacizumab) intravitreal injections compared to standard therapy in subjects with choroidal neovascularisation secondary to age-related macular degeneration: clinical trial design

**DOI:** 10.1186/1745-6215-9-56

**Published:** 2008-10-14

**Authors:** Praveen J Patel, Catey Bunce, Adnan Tufail, the ABC Trial Investigators

**Affiliations:** 1 Moorfields Eye Hospital, 162 City Road, London, UK

## Abstract

**Background:**

The management of neovascular age-related macular degeneration (nAMD) has been transformed by the introduction of agents delivered by intravitreal injection which block the action of vascular endothelial growth factor-A (anti-VEGF agents). One such agent in widespread use is bevacizumab which was initially developed for use in oncology. Most of the evidence supporting the use of bevacizumab for nAMD has come from interventional case series and this clinical trial was initiated because of the increasing and widespread use of this agent in the treatment of nAMD (an off-label indication) despite a lack of definitive unbiased safety and efficacy data.

**Methods and design:**

The Avastin^® ^(bevacizumab) for choroidal neovascularisation (ABC) trial is a double-masked randomised controlled trial comparing intravitreal bevacizumab injections to standard therapy in the treatment of nAMD. Patients are randomised to intravitreal bevacizumab or standard therapy available at the time of trial initiation (verteporfin photodynamic therapy, intravitreal pegaptanib or sham treatment). Ranibizumab treatment was not included in the control arm as it had not been licensed for use at the start of recruitment for this trial. The primary outcome is the proportion of patients gaining ≥ 15 letters of visual acuity at 1 year and secondary outcomes include the proportion of patients with stable vision and mean visual acuity change.

**Discussion:**

The ABC Trial is the first double-masked randomised control trial to investigate the efficacy and safety of intravitreal bevacizumab in the treatment of nAMD. This trial fully recruited in November 2007 and results should be available in early 2009. Important design issues for this clinical trial include (a) defining the control group (b) use of gain in vision as primary efficacy end-point and (c) use of pro re nata treatment using intravitreal bevacizumab rather than continuous therapy.

**Trial registration:**

Current controlled trials ISRCTN83325075

## Background

Age-related macular degeneration (AMD) is the leading cause of visual loss in patients over the age of 50 years in Europe and North America [1]. There are 2 forms of the disease with dry AMD and wet AMD (neovascular or exudative AMD). Neovascular age-related macular degeneration (nAMD) is characterised by choroidal neovascularisation (CNV) and is responsible for 75% of visual loss due to AMD despite only accounting for 25% of all cases [2].

Historically the prognosis for patients with subfoveal nAMD has been poor with the established treatment of verteporfin photodynamic therapy (PDT) only showing modest efficacy in reducing visual loss in patients with well-defined (predominantly classic or classic no occult) sub-types of nAMD [3]. Until the introduction of new therapies, there was no effective treatment for poorly defined (minimally classic or occult) subtypes of nAMD.

One of the key mediators implicated in the pathogenesis of CNV in nAMD is vascular endothelial growth factor-A (VEGF). New treatments have targeted VEGF with the introduction of agents administered by injection into the vitreous cavity with high binding specificity to VEGF (anti-VEGF agents). There are three agents which block the action of VEGF-A currently in clinical use. These agents are administered by intraocular (intravitreal) injections with repeated injection necessary every 4–6 weeks depending on the agent.

The first drug developed and licensed for use was pegaptanib sodium (Macugen^®^, Pfizer Inc, New York, NY). This agent is an oligonucleotide with high binding specificity for the 165 isoform of VEGF. The pivotal phase III randomised controlled trial reported a benefit in stabilising vision over sham treatment (70% of patients lost less than 15 letters of visual acuity at 1 year in treated groups compared to 55% in sham treated group) but disappointingly only 6% of treated patients improved vision by 15 letters or more at one-year follow-up [4].

Another agent which has gained favour with ophthalmologists is bevacizumab (Avastin^®^, Genentech Inc., South San Francisco, CA). This full-length monoclonal antibody binds to and blocks the action of all isoforms of VEGF. It was initially developed as an intravenous agent in the treatment of metastatic colorectal cancer [5] before ophthalmologists reported promising results using bevacizumab as an intravitreous treatment for nAMD in case series [6,7]. It has gained popularity worldwide as a treatment option for nAMD due to the low drug cost of treatment when used as an intraocular agent. Despite the widespread off-label ocular use of bevacizumab, there have been no definitive prospective, double-masked, randomised, controlled trials investigating the safety and efficacy of intravitreal bevacizumab for the treatment of nAMD.

Ranibizumab (Lucentis^®^, Genentech Inc., San Francisco, CA) is an antibody fragment developed from the bevacizumab molecule with increased binding affinity for all isoforms of VEGF. It has been shown to be more efficacious than sham treatment (placebo) in treating minimally classic or occult CNV [8] and PDT [9] in treating predominantly classic CNV. Patients treated with monthly intravitreal injections of ranibizumab for 1–2 years showed better visual acuity outcomes than patients treated with sham or PDT (40–33% of ranibizumab treated patients improving visual acuity by 15 letters or more compared to 4% of sham treated and 6% of PDT treated patients). Ranibizumab was licensed for use in the treatment of nAMD after the start of recruitment for this study and so was not included as a comparator arm. Randomised controlled trials comparing bevacizumab to ranibizumab have commenced recruitment but are likely to take a further 2–3 years before they report outcome data. In addition it is important to consider that a 4 weekly dosing interval for both bevacizumab and ranibizumab is planned to be used in these trials, which, given that studies have suggested a 6 weekly dosing schedule may be adequate for intravitreal bevacizumab [10], eliminates one of the main potential advantages bevacizumab over ranibizumab – the need for less intravitreal injections and less intensive patient follow-up.

In 2005, the use of bevacizumab in the treatment of nAMD was increasing both in the USA and Europe as ophthalmologists had received early promising reports of efficacy from interventional case series with approximately 1/3 of patients improving vision. At that time, the only licensed agents in the treatment of subfoveal nAMD were PDT and intravitreal pegaptanib injections which though associated with reducing visual loss, had shown only modest efficacy in improving vision (only approximately 5–6% of patients improving vision with either of these agents in randomised controlled trials). In addition to perceived better efficacy, repackaging pharmacies provide intravitreal bevacizumab for a fraction of the cost of pegaptanib, verteporfin or ranibizumab. However there are no large scale prospective, double-masked, randomised controlled trials supporting the use of bevacizumab for nAMD and there was therefore a need to design a clinical trial to investigate the efficacy and safety of intravitreal bevacizumab injections to standard therapy (PDT or pegaptanib) in the treatment of nAMD.

The current trial is designed to investigate whether intravitreal bevacizumab injections are an effective and safe treatment for nAMD when compared to standard therapy. It will also provide exploratory data on the efficacy of bevacizumab when compared to either PDT, pegaptanib or sham treatment alone.

## Methods and design

**Figure 1:**
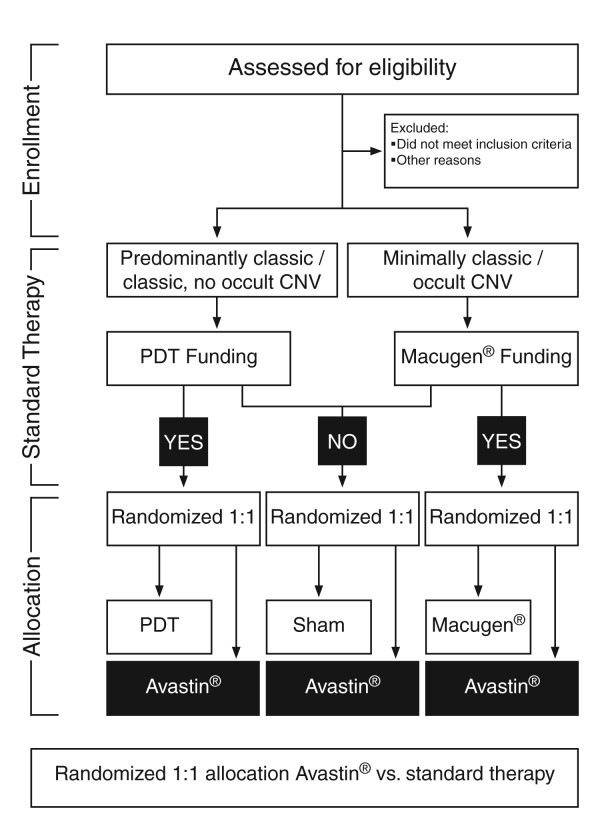
Summary of trial design.

Double-masked, randomised, controlled trial with 2 parallel treatment groups. Eligible patients were randomised in a 1:1 ratio to receive either intravitreal bevacizumab or standard therapy (either PDT, pegaptanib intravitreal injections or sham intravitreal injections). Only one eye per patient was included in the study and this was selected prior to randomisation. Standard therapy was determined prior to trial enrolment at which point patients were allocated to treatment groups by minimisation – a dynamic process which reduces the imbalance between trial arms with respect to standard treatment eligibility and site. During trial recruitment, patients with well-defined (classic, no occult or predominantly classic CNV) were funded for PDT in line with National Institute for Health and Clinical Excellence (NICE) guidance. However there was no national funding in place for patients with poorly defined forms of nAMD (minimally classic or occult CNV) as PDT had not shown efficacy over natural history for these lesion subtypes and the new anti-VEGF agents (pegaptanib and ranibizumab) had not been subject to appraisal by NICE. National Health Service (NHS) treatment was delivered on a case by case basis with many patients not funded for NHS treatment. Eligible patients with minimally classic or occult no classic CNV were either randomised to pegaptanib or sham treatment (based on funding of pegaptanib therapy). A summary of the enrolment and randomisation process in shown in Figure 1.

During the later stages of trial recruitment, after ranibizumab had been introduced into clinical practice, if patients were funded for ranibizumab, they were excluded from enrolment to this clinical trial. At the time of recruitment for this trial, since many patients who were refused public funding of treatment did not have medical insurance and could not afford the costs of private treatment, a significant proportion of patients remained untreated and therefore would consider involvement in a trial in which they were randomised to active treatment (intravitreal bevacizumab) or to a sham arm. Previous precedents for such active treatment versus sham or no treatment trial included the pegaptanib VISION trial [4] or the Lucentis MARINA trial [8]. The ABC trial fully recruited its patient population before final NICE guidance was issued on the use of pegaptanib or ranibizumab for treating nAMD in the NHS.

### Objectives

#### Primary

To test the hypothesis that in patients being treated for choroidal neovascularisation (CNV) due to age-related macular degeneration (nAMD), intravitreal bevacizumab injections can improve visual acuity (defined as a gain of ≥ 15 letters) in the treated eye in a greater proportion of patients than standard therapy after 12 months

#### Secondary

To test the hypothesis that in patients being treated for CNV due to age-related macular degeneration (nAMD):

1. Intravitreal bevacizumab injections are not associated with increased ocular and systemic adverse events compared to standard therapy after 12 months

2. Intravitreal bevacizumab injections can stabilise visual acuity (defined as a loss of <15 letters of visual acuity) in a greater proportion of patients than standard therapy after 12 months

3. Intravitreal bevacizumab injections can improve visual acuity (defined as a gain of ≥ 10 letters) in a greater proportion of patients than standard therapy after 12 months

4. Intravitreal bevacizumab injections can improve visual acuity in a greater proportion of patients than standard therapy at the 6 month time-point

5. Intravitreal bevacizumab injections can improve mean visual acuity when compared to standard therapy at the 12 month time-point

6. Intravitreal bevacizumab injections can lead to a greater reduction in macular thickness than in patients receiving standard therapy at the 6 and 12 month time-points

7. Intravitreal bevacizumab injections can lead to a greater reduction in leakage from CNV than in patients receiving standard therapy at the 12 month time-point

### Eligibility

#### Inclusion criteria

• Age >50 years

• Primary subfoveal CNV lesions secondary to AMD in the study eye

• An occult CNV must have presumed evidence of disease progression, defined as one or more of the following:

1. Deterioration of best corrected vision by 1 Snellen line or 5 letters on Early Treatment of Diabetic Retinopathy Study (ETDRS) charts within the past 3 months due to progression of CNV

2. Presence of sub or intraretinal blood

3. Growth of lesion size on fluorescein angiography by more than 10% in the past 3 months

• Evidence of central macular thickening assessed using optical coherence tomography (OCT)

• Total lesion size < 12 optic disc areas including all contiguous lesion components.

• Area of fibrosis < 25% of the total lesion area

• Area of subretinal blood less than 50% of total lesion area

• Best corrected visual acuity, using ETDRS charts of 6/12 to 6/96 (Snellen equivalent) in the study eye

Only one eye is assessed in the study. If both eyes are eligible, the one with the better visual acuity will be selected for treatment and study unless, based on medical reasons, the investigator deems the other eye the more appropriate candidate for treatment and study.

#### Exclusion criteria

Prior treatment with external-beam radiation therapy, transpupillary thermotherapy (TTT), thermal laser, or PDT in the study eye

Treatment with verteporfin in the non-study eye less than 7 days preceding Day 0,

• Previous participation in a clinical trial (for either eye) involving anti-angiogenic drugs (pegaptanib, bevacizumab, anecortave acetate, protein kinase C inhibitors, etc.)

• Previous intravitreal drug delivery (e.g., intravitreal corticosteroid injection or device implantation) in the study eye

• History of vitrectomy surgery in the study eye

• History of greater than mild non-proliferative diabetic retinopathy or any diabetic maculopathy

• History of retinal vascular occlusions (if considered likely to compromise potential for visual acuity improvement)

• History of glaucoma filtering surgery in the study eye

• History of corneal transplant in the study eye

• History of submacular surgery or other surgical intervention for AMD in the study eye

• Previous participation in any studies of investigational drugs within 1 month preceding Day 0 (excluding vitamins and minerals)

• Current or intending use of warfarin or known abnormal blood clotting

#### Lesion characteristics

• Subretinal haemorrhage in the study eye that involves the centre of the fovea, if the size of the haemorrhage is either >50% of the total lesion area or >1 disc area in size

• Subfoveal fibrosis or atrophy in the study eye.

• CNV in either eye due to causes other than AMD, such as ocular histoplasmosis, trauma, or pathologic myopia

• Retinal pigment epithelial tear involving the fovea in the study eye

#### Concurrent ocular conditions

• Any concurrent intraocular condition in the study eye (e.g., cataract or diabetic retinopathy) that, in the opinion of the investigator, could either require medical or surgical intervention during the 12 month study period to prevent or treat visual loss that might result from that condition, or if allowed to progress untreated, could likely contribute to loss of at least 2 Snellen equivalent lines of best corrected visual acuity over the 12 month study period

• Active intraocular inflammation (grade trace or above) in the study eye

• Current vitreous haemorrhage in the study eye

• History of rhegmatogenous retinal detachment or macular hole in the study eye

• History of idiopathic or autoimmune-associated uveitis in either eye

• Infectious conjunctivitis, keratitis, scleritis, or endophthalmitis in either eye

• Aphakia or absence of the posterior capsule in the study eye. Previous violation of the posterior capsule in the study eye is also excluded unless it occurred as a result of YAG posterior capsulotomy in association with prior, posterior chamber intraocular lens implantation.

• Spherical equivalent of the refractive error in the study eye demonstrating more than -8 diopters of myopia or signs of pathologic myopia with a refraction of 4–8 diopters

For subjects who have undergone prior refractive or cataract surgery in the study eye, the preoperative refractive error in the study eye cannot exceed -8 diopters of myopia.

• Intraocular surgery (including cataract surgery) in the study eye within 2 months preceding Day 0

• Uncontrolled glaucoma in the study eye (defined as intraocular pressure >30 mmHg despite treatment with anti-glaucoma medication)

#### Concurrent systemic conditions

• Premenopausal women not using adequate contraception

• History of other disease, metabolic dysfunction, or clinical laboratory finding giving reasonable suspicion of a disease or condition that contraindicates the use of an investigational drug or that might affect interpretation of the results of the study or render the patient at high risk for treatment complications

• Current treatment for active systemic infection

• Recent stroke, or cardiac event in the last 6 months, uncontrolled angina or hypertension.

#### Other

• History of allergy to fluorescein

• Inability to obtain fundus photographs or fluorescein angiograms of sufficient quality to be analyzed and graded by the central reading centre

• Inability to comply with study or follow-up procedures

All patients referred to the Trial Centres with active CNV due to AMD were informed of the clinical trial and were given patient information sheets. Those patients who were keen to participate were screened with those meeting the eligibility criteria invited to enrol. The study is conducted according to ICHGCP (International Conference on Harmonisation for Good Clinical Practice in clinical research), as set out in the European Union Clinical Trials Directive (2001) and associated UK Regulations (2004), which adhere to the principles of the Helsinki Declaration.

### Interventions

**Figure 2:**
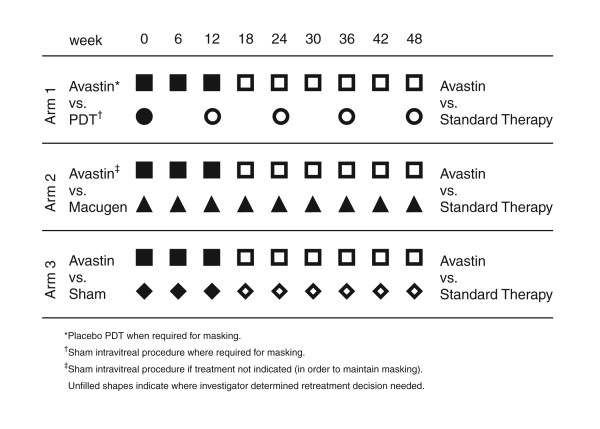
Summary of trial treatments.

Figure 2 summarises the trial treatments.

#### Active intervention. Intravitreal bevacizumab injections

Bevacizumab was administered by six weekly intravitreal injections (dose of 1.25 mg in 0.05 ml per injection) and a treatment strategy of 3 treatments with further injections as needed based on investigator driven retreatment with standardised retreatment criteria was used reflecting the real world use of this agent at the time of the trial (3 to 9 injections in total in 54 weeks). The bevacizumab injections were prepared by Moorfields Pharmaceuticals in line with UK pharmaceutical regulatory standards.

#### Control intervention. Intravitreal pegaptanib injections

These were given as per the product license with a dose of 0.3 mg of pegaptanib in 0.09 ml given as continuous therapy by intravitreal injection every 6 weeks for 1 year (9 injections in 54 weeks). This reflects the dosing and frequency of treatment used in the pivotal VISION randomised controlled trial reporting the efficacy and safety of pegaptanib in the treatment of nAMD [4].

#### Control intervention. Verteporfin photodynamic therapy

Verteporfin PDT is performed as described elsewhere [11]. Briefly, an unmasked trial member not involved in any outcome assessments and trained in the importance of maintaining masking prepares an infusion of verteporfin (6 mg/m^2 ^of body surface area) in 30 ml of 5% dextrose after calculating body surface area from a nomogram based on the height and weight of the patient. Intravenous infusion of the solution is given over 10 minutes after covering the infusion line and pump to maintain masking (the solution is green). Fifteen minutes after the start of the infusion, the unmasked ophthalmologist applies laser light (diode laser 689 nm) for 83 seconds (light exposure of 50 J/cm^2^, intensity 600 mW/cm^2^) to the CNV lesion through a fundus contact lens of known magnification. The laser spot size for each treatment is determined by measuring the greatest linear diameter of the CNV lesion on fluorescein angiography and adding an addition 1000 μm in order to provide an additional margin of 500 μm around the lesion. After the treatment the patient is advised to avoid bright sunlight for 48 hours to prevent possible photosensitive reactions.

#### Placebo treatment. Sham intravitreal injections

In line with previous randomised controlled trials [4,8], sham intravitreal injections rather than placebo intravitreal injections of vehicle are used. Sham injections are performed by following the procedure used to prepare the eye for injection but instead of an intravitreal injection, the hub of an empty 1 ml syringe is applied firmly to the conjunctiva to mimic an active injection. This procedure is ethically acceptable as it does not subject the patient to the potential risk of sight-threatening infection associated with intravitreal injections whilst maintaining masking by closely resembling an active injection procedure.

#### Measures to maintain masking

##### Placebo PDT

This is used for patients randomised to bevacizumab in cases in which standard care is PDT. The procedure is identical to that used for active verteporfin PDT except 5% dextrose is used as placebo with no verteporfin added. This is in line with previous studies [11]. Care was taken to ensure that the intravenous infusion pump and line were covered as the active verteporfin solution is green while the placebo infusion is a clear solution.

##### Additional use of sham injections

As the treatment of patients randomized to bevacizumab or pegaptanib involves the comparison of a pro re nata treatment (bevacizumab) to a treatment given continuously (nine, six weekly intravitreal injections of pegaptanib), to maintain masking, sham treatments were given to patients randomised to bevacizumab not requiring intravitreal treatment at that visit (weeks 18 to 48) based on the standardised retreatment criteria. This was to maintain masking as pegaptanib was delivered as continuous therapy (as per the product license) whereas bevacizumab was given as 3 initial injections with further retreatment as necessary using standardised retreatment criteria.

##### Evaluating the success of masking

Adequacy of masking was assessed by means of a questionnaire to patients and the masked investigator when the patient left the study to determine views on treatment allocation

### Outcome measures

#### Primary

1. Visual acuity score

An improvement in visual acuity is defined as a gain of 15 letters or more (3 lines) of best corrected visual acuity score at the 12 month time point compared with baseline, using ETDRS visual acuity charts and visual acuity measurement at a starting distance of 4 metres.

#### Secondary

1. Other visual acuity based outcomes

In addition to the conventional end-point of 15 or more letter gain in visual acuity, more recently a clinical trial visual acuity end-point of 10 or more letters has been suggested [12] and so the proportion of patients improving visual acuity using this end-point in both the bevacizumab treated and standard therapy treated group will also be described and analysed as a secondary outcome measure for the 6 month and 12 month time-points. Other alternative end-points of the proportion of patients gaining 5 or more letters and patient losing less than 15 letters of best corrected visual acuity score will also be examined similarly as secondary end-points. The mean change in visual acuity in the two groups at 12 months will also be reported.

2. Optical Coherence Tomography based retinal thickness measurement

Stratus OCT (Carl Zeiss Meditec, Inc) was used to obtain retinal thickness measurements using fast macular thickness mapping scan protocol. Radial line scanning (or cross-hairs when radial line scans not possible) was used to determine the presence or absence of macular fluid (intra-retinal cysts and sub-retinal fluid) at each visit. The mean change in macular thickness in the bevacizumab and standard therapy groups at the 6 and 12 month time-points will be reported.

3. Contrast sensitivity measurement

Pelli-Robson charts were used to measure contrast sensitivity [13].

4. Reading ability measurement

Minnesota Reading (MNREAD) charts [14] were used to assess reading ability with measurement of maximum reading speed, critical print size and reading acuity.

#### Adverse events

These will be documented and serious adverse events will be reported to the Medicines and Healthcare Products regulatory Agency (MHRA). Both ocular and systemic adverse events will be noted. There will also be a specific report of any adverse events meeting the Antiplatelet Trialists' Collaboration (APTC) criteria [15]. As this is a double-masked trial, adverse events will be reviewed by the Data Monitoring Committee (DMC) who may ask to be unmasked to treatment allocation.

### Visit schedule and assessments

Randomisation and treatment occur up to 14 days after a screening visit (or on the same day). After baseline treatment, patients attended again at week 1 for a safety visit (no treatment given). Further follow-up visits with repeat treatment occurred at week 6, 12, 18, 24, 30, 36, 42 and week 48. The study exit visit occurs at week 54 (1 year). This gives 9 treatment visits with the first 3 intravitreal bevacizumab treatments compulsory (baseline, week 6 and week 12) with further treatments as needed based on standardised retreatment criteria (at weeks 18, 24, 30, 36, 42, 48). Safety assessments consist of recording all adverse events, monitoring of blood pressure and pulse at every visit with blood tests at baseline, week 24 and study exit (week 54).

**Figure 3:**
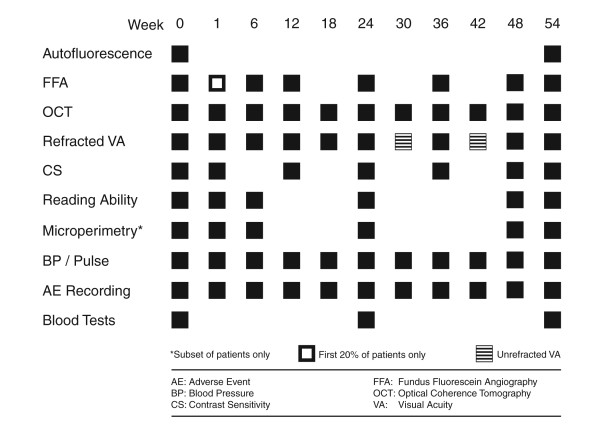
Summary of trial visits.

Study assessments include functional outcome measures including best-corrected visual acuity assessments, contrast sensitivity measurement and measurement of reading ability (using MNREAD acuity charts). Structural outcome was assessed at every visit using OCT measures of retinal thickness and qualitative features of CNV activity. Fundus fluorescein angiography was performed at baseline and weeks 6, 12, 24, 36 48 and week 54 visits (with additional fluorescein angiography at week 1 for the first 20% of patients) to allow assessment of any change in CNV size and leak. The trial visits are summarised in Figure 3.

### Trial size

A Fisher's exact test with a 0.050 two-sided significance level will have 90% power to detect the difference between a Group 1 proportion, π_1_, of 0.300 and a Group 2 proportion, π_2_, of 0.060 when the sample size in each group is 57. To allow for a potential 12% loss to follow-up we plan to recruit 65 patients per treatment group. (Previous clinical trials for nAMD [4,11], suggest that upto 10% of patients may be lost to follow-up during the trial). With these numbers we would have 82% power to detect differences of 0.25 and 0.06.

### Statistical analyses

Baseline characteristics of patients in each treatment arm will be summarised. The proportion of patients who gain 15 letters of best corrected visual acuity (BCVA) or more in each group at 12 months will be provided with 95% confidence intervals computed by the exact binomial method. A Fisher's exact test will be conducted to assess whether or not any observed difference in these proportions is statistically significant. Odds ratios will be reported together with 95% confidence intervals to illustrate the contrast between bevacizumab and standard treatment. If any imbalance in prognostic factors is detected, logistic regression will be conducted to assess the impact of any imbalance and adjusted odds ratios reported. Data will be analysed according to the group to which patients were originally assigned (i.e. intent to treat). In addition to the intention to treat analysis, a per protocol analysis will be carried out.

The proportion of patients in each group who at 6 and 12 months have

a) gained 5 letters of BCVA

b) gained 10 or more letters of BCVA

c) lost fewer than 15 letters of BCVA

will be reported with 95% confidence intervals together with a description of the average change in OCT determined central macular thickness and BCVA at 12 months. Comparative results (odds ratios and mean differences) will also be reported with 95% confidence intervals. These form part of the secondary end-points for the trial.

Kaplan Meier plots will be constructed to examine rates of improvement of BCVA and Cox regression or log rank tests conducted. Adherence to the proportional hazards assumption will be assessed.

Adverse events will be tabulated by treatment group.

### Trial organisation

The trial centres and investigators are listed in Table 1

**Table 1:** Trial management

Trial Operations Committee	
Adnan Tufail	Chief Investigator, Moorfields Eye Hospital, London, UK
Praveen J Patel	Principal Investigator, Moorfields Eye Hospital, London, UK
	
Laura Henderson (Mrs Ola Segun-Odumosu from March 2008)	Trial Manager, Moorfields Eye Hospital, London, UK
	

Trial Steering Committee	

Astrid Fletcher	Professor of Epidemiology and Ageing, London School of Hygiene and Tropical Medicine, London, UK
Adnan Tufail	Chief Investigator, Moorfields Eye Hospital, London, UK
Catey Bunce	Trial Statistician, Moorfields Eye Hospital, London, UK
Praveen J Patel	Principal Investigator, Moorfields Eye Hospital, London, UK
Richard Wormald	Consultant Ophthalmologist, Moorfields Eye Hospital, London, UK
	

Data Monitoring Committee	

Catey Bunce	Trial Statistician, Moorfields Eye Hospital, London, UK
Marion Campbell	Professor of Health Services Research, University of Aberdeen, UK
Robyn Guymer	Professor of Ophthalmology, University of Melbourne, Australia
Frank G. Holz	Professor of Ophthalmology, University of Bonn, Germany

#### Trial Steering Committee

The Trial Steering Committee monitor and supervise the trial and comment on any proposed major protocol amendments (Table 1).

#### Data Monitoring Committee

The data monitoring committee (DMC) includes 2 independent ophthalmologists with a specialist interest in retinal disease and an independent statistician with clinical trials experience (Table 1). No formal interim analysis is planned. The trial statistician will report to an independent DMC which will monitor the trial in all its respects. It will review safety data on a monthly basis and if appropriate will conduct an unmask safety analysis.

#### Trial Operations Committee

An operations committee consisting of the Chief Investigator, the Principal Investigator at the co-ordinating centre and the Trial Manager meet every week. During these meetings, the committee reviews the progress of the study identifying any problems or issues at all 3 sites.

#### Trial co-ordination

The trial is centrally co-ordinated from the Clinical Trials Unit (CTU) at Moorfields Eye Hospital. This provides the telephone randomisation service (which uses minimisation) and is responsible for data management.

### Trial documentation and data collection

All trial centres are supplied with a Protocol, Standard Operating Procedures guidance, Source Documentation and Case Report Forms. Serious adverse events are reported to Moorfields Eye Hospital (the Trial Sponsor) and to the MHRA.

### Ethics and competent authority review

Applications to UK Main and Local Research Ethics Committees (REC) have received favourable opinions and a Clinical Trials Authorisation has been issued by the MHRA.

### Publication policy

The results of this trial will be submitted for publication to peer-review medical journals regardless of whether the outcome is in favour of the trial intervention.

### Trial timetable

#### Trial start

August 2006

#### Trial recruitment completed

November 2007

#### Trial end

December 2008

#### Trial duration

2 years, 5 months

#### Duration of each patient's participation

1 year (54 weeks)

## Discussion

Since the first reports of the efficacy of bevacizumab in treating nAMD, there has been increasing use of this drug for this unlicensed indication and bevacizumab is probably the most widely used drug worldwide to treat nAMD because of its low unit cost despite the availability of alternative licensed therapies. However, most of the evidence supporting its use comes from interventional case and in this age of evidence-based medicine, there is no data from prospective, double-masked, multi-centre randomised controlled trials comparing the efficacy and safety of intravitreal bevacizumab to other therapies in the treatment of nAMD. The Avastin^® ^(bevacizumab) for CNV trial will be the first definitive, prospective, double-masked, multi-centre, randomised controlled trial reporting unbiased efficacy and safety data for intravitreal bevacizumab in the treatment of nAMD with results expected in early 2009.

In addition, the trial will provide information about the effect of treatment on structural (qualitative and quantitative OCT measures) and other functional outcome measures (including contrast sensitivity and reading ability). Exploratory analyses including possible prognostic indicators will also be of interest.

The Avastin^® ^(bevacizumab) for CNV trial incorporates several novel features in the trial design. In contrast to previous multi-centre randomised controlled trials in the field [3,4,8,9], this study is the first to use visual gain as the primary outcome measure rather than stabilisation in vision with secondary outcomes including mean visual acuity change to maximise use of data points. This shift from stabilisation to visual gain as the primary end-point reflects the changing expectation of both patients and physicians in the treatment of nAMD.

Treatment in the comparator or control arm is determined by funding of standard therapy. This may be viewed as both a strength and a weakness of the study. Though not allowing comparison with a single agent, the choice of a comparator arm with one of 3 different treatments (2 active and one sham) reflects the usual treatment of patients in the NHS at the time of recruitment. It does not undermine the analysis of results as all treatments in the comparator arm have shown to offer patients a similar chance of improvement in vision (a maximum proportion of 0.06 improving vision with either comparator treatment at one year [3,4]). Ranibizumab was not one of the therapies included in the comparator arm as this drug only reached market after the start of recruitment and was not widely available during the recruitment phase of this trial.

Another novel design feature is the use of pro re nata treatment with bevacizumab (based on investigator determined retreatment with standardised retreatment criteria) after three initial treatments. This approach reflects clinical practice with clinicians using retreatment criteria to determine further treatment with anti-VEGF agents and should make the results more translatable into clinical practice. However this is distinct from the approach taken in the pivotal anti-VEGF agent trials in which a continuous treatment strategy was used. The use of continuous pegaptanib (reflecting the product license for pegaptanib) in the comparator arm is identical to the dosing used in the phase III trial comparing pegaptanib to sham treatment [4].

The ABC trial is the first multi-centre randomised controlled trial to use OCT in all patients both using retinal thickness data to assess outcome and to use qualitative interpretation of scans in deciding on retreatment of patients with AMD. Previous randomised trials have used OCT only in a sub-set of patients with no use of OCT based retreatment criteria.

In summary, the ABC trial uses a pragmatic yet high-quality trial design to evaluate the efficacy of intravitreal bevacizumab in the treatment of nAMD. The treatment strategy used reflects the clinical use of bevacizumab in treating nAMD and should help clinicians in translating the results from the trial into clinical practice. The trial aims to augment the evidence base for therapeutic options in the treatment of nAMD.

## Competing interests

The authors declare that they have no competing interests.

## Authors' contributions

PJP participated in development of the trial protocol and the trial design, coordinated the trial's set-up at Moorfields Eye Hospital and facilitated the set-up of the other study sites in London; prepared the trial's manual of operations, study documentation and publicity and drafted the manuscript. CB participated in development of the trial protocol, setup the trial's randomisation procedure and coordinates statistical analyses. AT conceived and designed the trial, secured trial funding and led its set-up at Moorfields Eye Hospital and helped to draft the manuscript.
